# Effects of resistance training on subjective sleep quality, excessive daytime sleepiness, and quality of life in patients with obstructive sleep apnea: a secondary analysis from a randomized controlled trial

**DOI:** 10.1007/s11325-026-03842-5

**Published:** 2026-10-20

**Authors:** José Ricardo Vieira de Almeida, Daniela Karina da Silva Ferreira, Victória Duarte de Arantes Lima, Enrique Nascimento Nazário dos Santos, Nadja Sylmara dos Santos de Oliveira Silva, José Lucas Porto Aguiar, Elton Carlos Felinto dos Santos, Antônio Henrique Germano-Soares, Rodrigo Pinto Pedrosa, Andrea Maculano Esteves, Breno Quintella Farah, Ozeas de Lima Lins-Filho

**Affiliations:** 1 Graduate Program in Physical Education, Federal University of Pernambuco, Av. dos Reitores. S/N Cidade Universitária, Recife, Brazil; 2 Department of Physical Education, Federal University of Pernambuco, Recife, Brazil; 3 Graduate Program in Health Sciences, University of Pernambuco, Recife, Brazil; 4 Department of Physical Education, Faculdade Pernambucana de Saúde, Recife, Brazil; 5 Sleep and Heart Laboratory, Pronto Socorro Cardiológico de Pernambuco (PROCAPE), University of Pernambuco, Recife, Brazil; 6 Laboratory of Sleep and Exercise, School of Applied Sciences, State University of Campinas, Limeira, Brazil; 7 Graduate Program in Movement Sciences, Federal Rural University of Pernambuco, Recife, Brazil

**Keywords:** Resistance training, Obstructive sleep apnea, Sleep, Daytime somnolence

## Abstract

**Purpose:**

This study aimed to verify the effect of eight weeks of resistance training (RT) on subjective sleep quality, daytime sleepiness, and quality of life in adults with OSA.

**Methods:**

In this randomized controlled clinical trial, 25 adults with moderate to severe OSA (age: 54.6 ± 7.2 years; BMI: 36.1 ± 7.9 kg/m²; apnea-hypopnea index: 52.2 ± 27.4 events/hour) were included. Participants were randomized into an RT group (three sessions per week) or a control group [CG (two sessions per week)]. Sleep quality, excessive daytime sleepiness, and quality of life were assessed using the Pittsburgh Sleep Quality Index (PSQI), Epworth Sleepiness Scale (ESS), SF-36, respectively. Generalized estimated equations verified differences between groups over time.

**Results:**

Differences were observed in domains sleep latency (RT: Δ -1.0 ± 0.3 vs. CG: 0.2 ± 0.2; *p* = 0.018), sleep duration (RT: Δ -0.6 ± 0.3 vs. CG: -0.2 ± 0.2; *p* = 0.012), sleeping medication (RT: Δ -0.2 ± 0.1 vs. CG: 0.8 ± 0.3; *p* = 0.007), and overall PSQI score (RT: Δ -3.2 ± 0.6 vs. CG: 0.2 ± 0.8; *p* = 0.004). Regarding SF-36, differences were found in emotional aspects (RT: Δ 29.6 ± 14.8 vs. CG: -17.9 ± 10.0; *p* = 0.008) and global score (RT: Δ 10.8 ± 3.3 vs. CG: -3.4 ± 3.1; *p* = 0.002) were observed. No difference was observed in ESS.

**Conclusion:**

An eight-week RT program improves subjective sleep quality and quality of life in adults with moderate to severe OSA. Clinical trial registration (Brazilian Clinical Trials Registry [ReBec]): # RBR-2rg97kz. Date of registration: 01/17/2025.

## Introduction

The obstructive Sleep Apnea (OSA) affects nearly 30% of individuals worldwide [1] and is associated with a significant worsening of sleep quality, increased daytime sleepiness [2, 3], and worsened quality of life [4]. Accordingly, improving these self-perceived outcomes is cornerstone of OSA management and exercise training has been shown to be effective in improving the sleep-related subjective sleep quality and excessive daytime sleepiness [5] in addition to improving in quality of life [5] being suggested as adjunct therapy for patients with OSA [6].

Although aerobic training isolated or combined with resistance training (RT) are the most studied exercise programs showing beneficial effects in those subjective OSA-related outcomes [5, 6] a previous study by our group, found that eight weeks of RT alone improved OSA severity and reduced arousals and abdominal obesity [7]. However, the primary analysis of this clinical trial focused on the severity of OSA, excluding patient-reported outcomes such as subjective sleep quality, excessive daytime sleepiness, and quality of life, which remain poorly explored. To address this gap, this secondary analysis aimed to investigate the effect of an eight-weeks resistance training program on subjective sleep quality, excessive daytime sleepiness, and quality of life in individuals with obstructive sleep apnea.

## Methods

This randomized controlled clinical trial with a parallel-group design was conducted over eight weeks. The study received ethical approval from the Research Ethics Committee of the University of Pernambuco (# 6.989.235) and adults with moderate to severe OSA were allocated to resistance training group (RT) or control group (CG), with pre- and post-interventions assessment.

The inclusion criteria were diagnosis of moderate to severe OSA (apnea-hypopnea index ≥ 15 events/hour) assessed through complete nocturnal polysomnography (PSG; Brain Wave II, Neurovirtual) [8], absence of prior OSA treatment, and adequate physical condition to perform RT without contraindications. Exclusion criteria included failure to complete at least 80% of sessions and initiation of any OSA-related therapy. Anthropometric assessments comprised neck, waist, hip, and calf circumferences (cm), body weight (kg), and height (cm). Body composition (fat mass and fat-free mass) was evaluated by 8-point tetrapolar bioelectrical impedance analysis with tactile electrodes.

Subjective sleep quality was assessed with the Pittsburgh Sleep Quality Index (PSQI) [9]. Each domain was calculated in isolation [10] and together to compute the overall score which ranges from 0 to 21 points with values between 0 and 5 points indicating “good sleep quality”, between 6 and 10 indicating “poor sleep quality”, and scores > 10 points indicating “sleep disturbance” [11]. Excessive daytime sleepiness (EDS) was evaluated with the Epworth Sleepiness Scale (ESS) [12]. Each item is scored from 0 to 3 and overall score ≥ 10 indicated presence of EDS. Quality of life was evaluated using the Short-Form Health Survey (SF-36) [13]. The SF-36 assesses eight domains: physical functioning, role limitations due to physical problems, bodily pain, general health perceptions, vitality, social functioning, role limitations due to emotional problems, and mental health. Each domain ranges from 0 to 100.

Resistance training group (RT) consisted of three weekly sessions over eight weeks (24 sessions) with eight exercises for upper and lower body, whereas control group consisted of two weekly sessions over the same period with stretching exercises for thirty minutes. The RT and CG protocols have been previously published [7]. Generalized estimating equations were used to detect between-group differences, pre-intervention to post-intervention changes, and group x time interactions for the outcomes. The significance level was set at P less than 0.05 for all analysis.

## Results

**Fig. 1:**
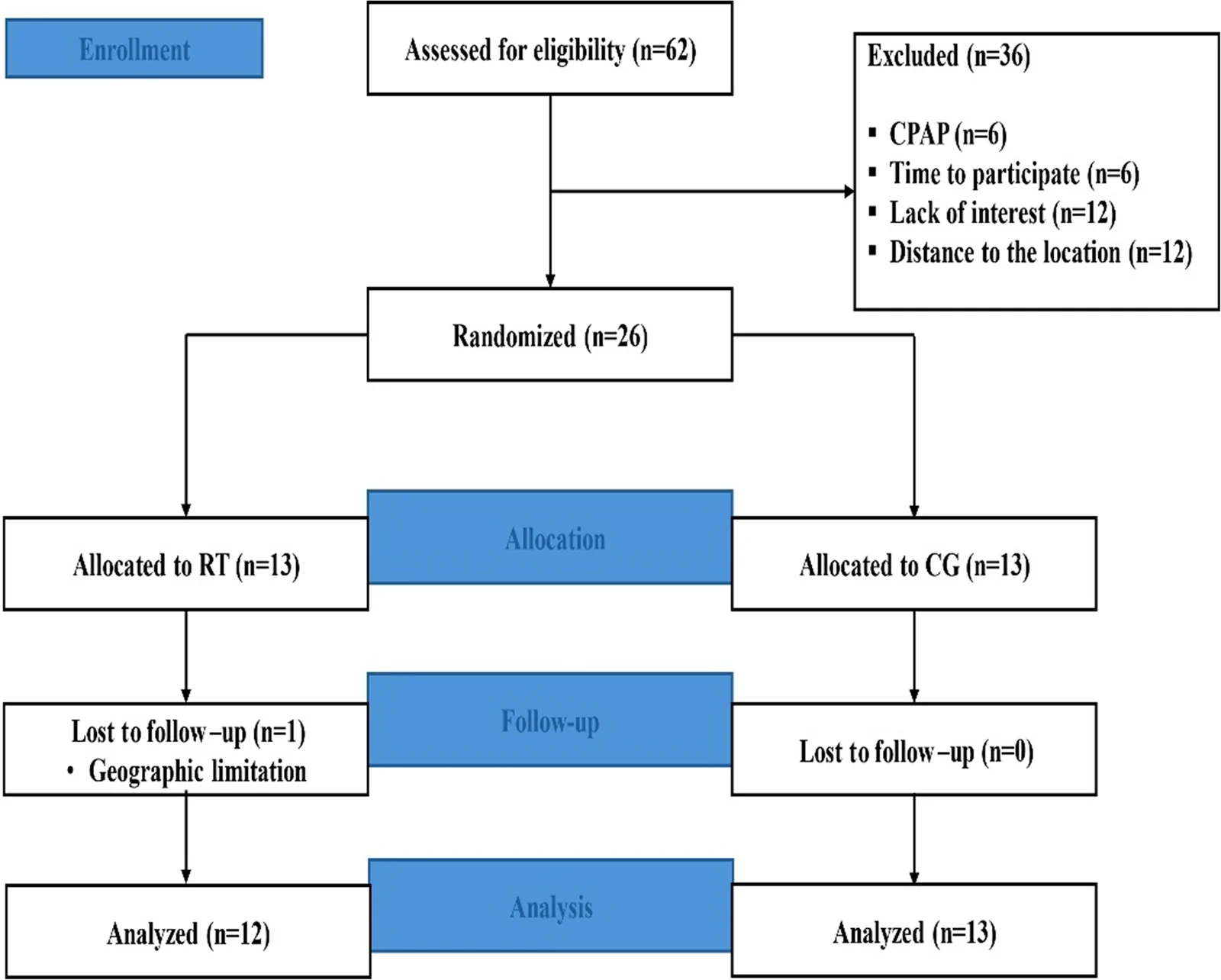
Study flowchart

Twelve participants in RT and 13 in CG completed the study (Fig. 1). Table 1 presents the anthropometric and sociodemographic characteristics of the participants. After the intervention, there was a time x group interaction in the AHI with a difference between-groups of 17.4 ± 7.2 events/hour (RT from 50.9 ± 6.9 to 30.0 ± 7.3; CG from 51.9 ± 7.7 to 48.5 ± 7.6 e/h, *p* = 0.015) [7].

**Table 1:** Anthropometric and sociodemographic variables in both RT and CG groups

	RT (*n* = 12)	CG (*n* = 13)	*p*
Age, years	55.2 ± 6.4	54.1 ± 8.1	0.710
Male, n (%)	7 (58.3)	6 (46.2)	0.695
Body weight, kg	103.1 ± 19.4	91.9 ± 19.4	0.155
Body mass index, kg/m^2^	38.1 ± 9.1	33.8 ± 5.2	0.150
Neck circumference, cm	42.2 ± 6.2	40.6 ± 4.5	0.456
Waist circumference, cm	119.1 ± 15.8	110.6 ± 8.8	0.108
Hip circumference, cm	121.7 ± 19.4	115.8 ± 18.2	0.439
Body fat, %	40.8 ± 10.2	38.1 ± 7.5	0.521
Fat free mass, %	59.0 ± 12.5	55.6 ± 10.4	0.514
Smoker, %	-	7.7	0.141
Hypertension, %	33.3	26.9	0.420
Diabetes mellitus, %	16.7	7.7	0.619
Medications			
ACEI, %	16.7	7.7	0.490
ARB, %	41.7	23.1	0.319
Thiazides/loop diuretic, %	41.7	30.8	0.568
Beta-blocker, %	16.7	23.1	0.689
Calcium channel blocker, %	16.7	38.5	0.225
Oral hypoglycemic agents, %	16.7	7.7	0.619

*CG* control group, *RT* resistance training, *ACEI*  angiotensin-converting enzyme inhibitor, *ARB* angiotensin receptor blocker. Independent Samples T test

**Fig. 2:**
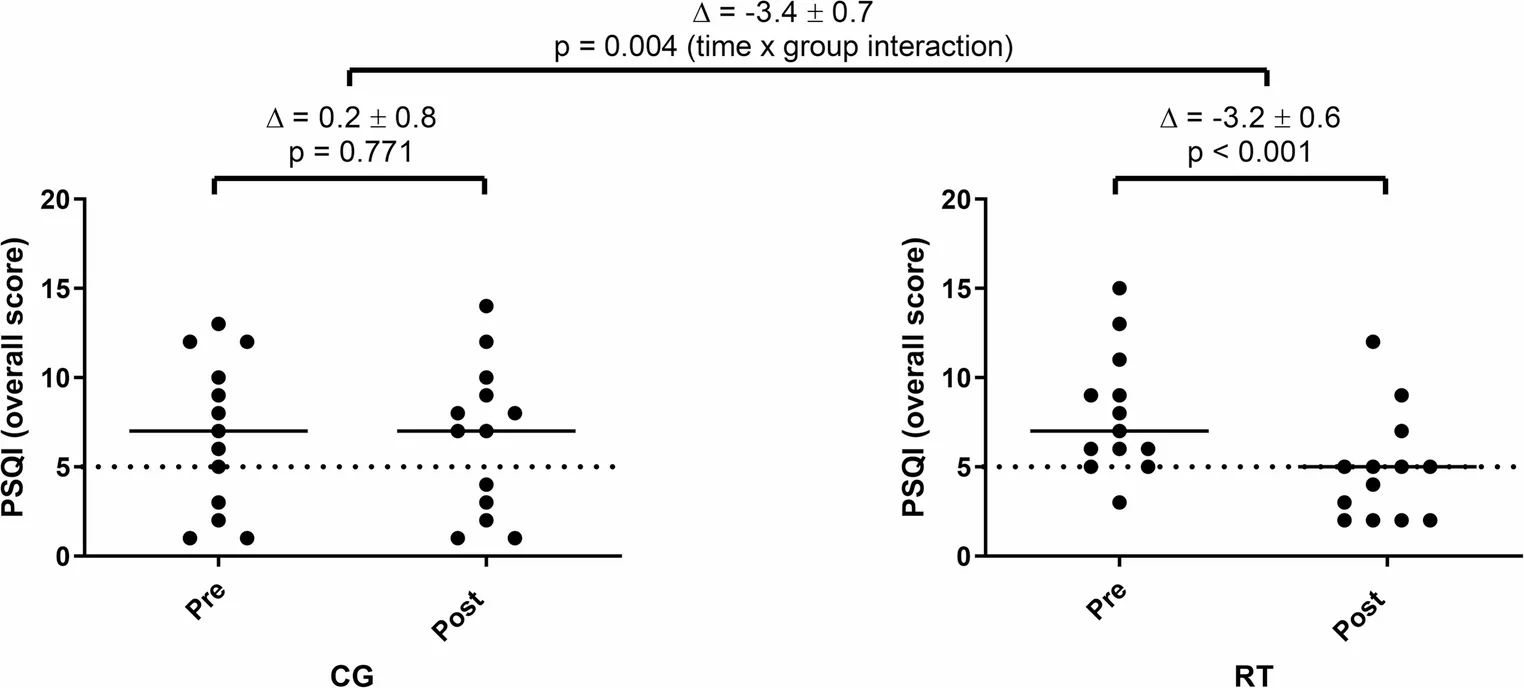
PSQI global scores before and after the 8-week interventions in the controlgroup (CG) and resistance training group (RT). The dashed line indicates the cutoff score of 5. Data were analyzed using generalized estimating equations

A significant time × group interaction was observed for the PSQI global score (*p* = 0.004; Fig. 2), which decreased in the RT group (7.9 ± 0.9 to 4.8 ± 0.8; *p* < 0.001), but not in the CG (6.9 ± 1.1 to 6.6 ± 1.1; *p* = 0.771). The effects of RT on PSQI domains, EDS, and quality of life are shown in Table 2. Significant interactions were found for sleep latency (*p* = 0.018), sleep duration (*p* = 0.012), and sleep medication (*p* = 0.007), while no between-group difference was observed for EDS.

**Table 2:** Subjective sleep domains, excessive daytime sleepiness, and quality of life at pre- and post-RT and CG interventions

	RT (12)		CG (13)		*P* (time x group)
	Pre	Post	Pre	Post	
PSQI domains					
Sleep quality, points	1.3 ± 0.2	1.0 ± 0.2	1.5 ± 0.3	1.3 ± 0.1	0.571
Sleep latency, points	1.5 ± 0.3	0.5 ± 0.2^*^	1.2 ± 0.3	1.1 ± 0.3	**0.018**
Sleep duration, points	0.9 ± 0.3	0.3 ± 0.1^*^	0.6 ± 0.3	0.9 ± 0.3	**0.012**
Sleep efficiency, points	1.1 ± 0.3	0.8 ± 0.3	1.5 ± 0.3	1.0 ± 0.3	0.584
Sleep disturbance, points	1.1 ± 0.2	0.6 ± 0.2^*^	1.0 ± 0.2	0.9 ± 0.9	0.266
Sleep medications, points	0.7 ± 0.4	0.5 ± 0.3	0.0 ± 0.0	0.8 ± 0.3^*^	**0.007**
Daytime dysfunction, points	1.3 ± 0.8	1.0 ± 0.2	1.0 ± 0.2	0.8 ± 0.2	0.819
Epworth Sleepiness Scale					
Excessive Daytime Sleepiness, points	7.1 ± 1.4	5.9 ± 1.1	9.6 ± 1.6	9.8 ± 1.3	0.372
Quality of Life					
Overall quality of life, points	56.2 ± 3.7	66.9 ± 4.4*	68.5 ± 5.0	65.1 ± 6.6	**0.002**
Functional capacity, points	50.8 ± 7.2	56.9 ± 6.2	71.5 ± 5.0	66.2 ± 5.2	0.201
Social aspects, points	83.4 ± 6.1	77.0 ± 6.0	82.9 ± 6.2	79.5 ± 6.1	0.137
Mental health, points	72.9 ± 4.4	80.2 ± 4.1	75.1 ± 6.4	76.0 ± 6.8	0.308
Body pain, points	64.0 ± 7.9	64.6 ± 9.7	68.4 ± 7.9	66.7 ± 8.2	0.817
Physical aspects, points	38.5 ± 10.8	66.2 ± 12.3	67.3 ± 11.7	63.5 ± 12.4	0.101
Emotional aspects, points	53.8 ± 12.3	83.4 ± 7.8^**^	84.6 ± 10.0	66.7 ± 12.0	**0.008**
General health	45.4 ± 4.5	42.3 ± 5.3	45.4 ± 4.4	45.0 ± 5.2	0.664
Vitality, points	46.9 ± 6.4	58.5 ± 6.4	52.7 ± 7.1	56.9 ± 7.2	0.334

*PSQI* Pittsburgh Sleep Quality Index. Generalized Estimating Equations test

*p<0.05 pre vs. post and *p<0.01 pre vs. post

A significant time × group interaction was also found for the SF-36 overall score, which increased in the RT group (56.2 ± 3.7 to 66.9 ± 4.4; *p* = 0.002), but not in the CG (68.5 ± 5.0 to 65.1 ± 6.6). Emotional role limitations also showed a significant interaction (*p* = 0.008), increasing in the RT group (53.8 ± 12.3 to 83.4 ± 7.8; *p* < 0.05) and decreasing in the CG (84.6 ± 10.0 to 66.7 ± 12.0).

## Discussion

Our study demonstrated that eight weeks of RT improved sleep latency, sleep duration, sleep medication, overall sleep quality, emotional aspects, and overall score of quality of life. OSA affects sleep duration and quality [14], causes EDS [15], and worsens quality of life [16], and, although these outcomes are improved by CPAP [17], its long-term low adherence with near 40% of adults using 4 h or more per night, highlights the importance for alternative approaches such as physical exercise [7].

In the present study, the RT improved overall sleep quality by reducing the PSQI score by 3.2-points, which is comparable to the ~ 3.5-point reduction previously reported by our group [10]. Although evidence on the effects of RT on overall sleep quality remains limited [9], the 1-point reduction described in the only previous study is directionally consistent with our findings. Although we cannot state, it is plausible to speculate that this improvement was partially mediated by a reduction in the number of arousals following RT [7], as previous evidence has shown that the number of microarousals is associated with overall PSQI [18]. In addition, given that sleep quality is linked to the pathophysiology of OSA, the reductions in AHI [7] may also explain the improvement observed in global sleep quality.

The improvement in sleep latency indicated that the sleep patterns of adults with OSA were positively affected by RT. The 1-point reduction in the sleep latency is comparable to a 0.8-points reduction observed in other forms of exercise training [10]. Although this domain was not previously addressed with RT in adults with OSA, RT reduces objective sleep latency in adults with other clinical conditions that affect sleep [19, 20]. Moreover, the reduction found in the present study aligns with the findings of the objectively assessed sleep latency in the Sa Souza’s study [19], suggesting that it might reflect in the perception of the sleep latency in RT group.

A 0.6-points reduction in the sleep duration was observed in the present study. Although there are no studies addressing the effects of RT program on the subjective sleep duration in adults with moderate to severe OSA, this improvement has been found with RT in elderly with other sleep disturbances [21]. Considering the reduction in the sleep latency, it indicates that the feeling of less time to fall asleep might have reflected in perception of longer sleep duration.

The differences found in the sleep medication after the intervention period was driven by an increase in medication use in the CG rather than by a reduction in the RT. Although pharmacological therapy is not the first-line treatment for OSA, due to the lack of awareness regarding non-pharmacological management options, individuals often rely on medications that provide immediate but short-term effects on sleep. Thus, although we did not control for medication use, the CG may have increased their use of sleep medications over the intervention period.

Although no previous study has investigated the effects of RT on the quality of life of adults with OSA, improvements in the emotional domains and overall quality of life were found. The RT has been associated with increased muscle strength and physical performance [22], and these physiological adaptations may contribute to a greater ability to perform activities of daily living, being particularly relevant for individuals with OSA, who frequently experience fatigue and reduced functional capacity [23]. Furthermore, it has been observed that RT improves symptoms related to emotional aspects, including anxiety and depression, in other clinical conditions [24]. Moreover, over 40% of patients in RT were under diuretic therapy, which could have contributed for the improvement in quality of life [25].

Agreeing with Sá-Sousa’s study [19], no significant changes were observed in EDS. This suggests that improvements in EDS may not occur consistently with exercise interventions, even when positive effects are seen in other sleep-related parameters. Daytime sleepiness is a multifactorial symptom not determined solely by apnea severity, as in adults, the EDS intensity often does not correlate with AHI, suggesting that other factors such as sleep fragmentation [15, 26] may contribute to EDS [27], regardless OSA severity.

Some limitations should be highlighted in the present study, such as the small sample size, the short duration, and the limited generalizability of the results to broaden OSA populations. Sample size calculation was based on the AHI; thus, the current study may have had inadequate statistical power for the primary outcomes. Moreover, adults were middle-aged in the present study, thus, the results cannot be extrapolated for older population. Therefore, future research should involve larger sample sizes and with broader age range.

In conclusion, eight weeks of RT significantly improved perceived sleep quality and quality of life in untreated patients with moderate-to-severe OSA.

## Data Availability

The datasets generated during and/or analyzed during the current study are available from the corresponding author on reasonable request.
